# Effect of Computer Tasks in Straight Gaze on Corneal Topographic Indices

**DOI:** 10.18502/jovr.v19i2.12737

**Published:** 2024-06-21

**Authors:** Zeinab Tanhaye Shamsabady, Asieh Ehsaei, Monireh Mahjoob

**Affiliations:** ^1^Department of Optometry, Mashhad University of Medical Sciences, Mashhad, Iran; ^2^Refractive Errors Research Center, Mashhad University of Medical Sciences, Mashhad, Iran; ^3^Health Promotion Research Center, Department of Optometry, Rehabilitation Sciences Faculty, Zahedan University of Medical Sciences, Zahedan, Iran; ^5^Zeinab Tanhaye Shamsabady: https://orcid.org/0000-0002-9080-8534; ^6^Monireh Mahjoob: https://orcid.org/0000-0001-9455-0721

**Keywords:** Cornea, Corneal Aberration, Corneal Tomography, Corneal Topography, Pentacam

## Abstract

**Purpose:**

To investigate the effect of the near visual task of movie-watching in the straight gaze position on corneal topographic parameters.

**Methods:**

Thirty myopic young adults with an average age of 25.10 ± 4.13 years were recruited for the study. The designed near visual task consisted of watching a movie in the straight gaze position at a distance of 40 cm for 30 minutes. Corneal topography was performed using Oculus Keratograph 4 (OCULUS, Wetzlar, Germany) before and immediately after watching the movie, as well as at 10, 20, and 30 minutes intervals after completing this near visual task. Zernike coefficients, asphericity indices, refractive parameters, and diagnostic indices of keratoconus were recorded for statistical analysis.

**Results:**

Movie-watching at a close distance solely using the straight gaze position had no effect on Zernike coefficients (*P* > 0.130). Also, watching the movie had no effect on other corneal topography parameters including irregularities (*P *= 0.208), spherical eccentricity (*P *= 0.270), maximum decentration (*P* = 0.553), axis of maximum decentration (*P *= 0.186), peripheral astigmatism (*P *= 0.179), and average asphericity of the quadrants at 10 to 30º (*P* > 0.163).

**Conclusion:**

The results of the present study showed that watching movies in the straight gaze position had no effect on corneal topographic parameters and did not cause errors in corneal topographic measurements.

##  INTRODUCTION

The cornea which has a significant impact on the optical characteristics of the eye, accounts for almost two-thirds of the refractive power of the eye.^[1]^ Therefore, slight changes in the cornea can affect refraction,^[2]^ aberrations,^[3]^ and consequently the quality of retinal images. The accuracy and stability of corneal topography are important in many clinical cases, including contact lens fitting,^[4]^ refractive surgeries,^[5,6]^ corneal transplantation,^[7]^ diagnosis and follow-up of keratoconus,^[8]^ orthokeratology lens fitting,^[9]^ and implantation of aspheric intraocular lenses.^[10]^ Various factors such as gender, race, age, eyelid shape and position, and palpebral ﬁssure can alter short-term and long-term stability of the shape of the cornea.^[11,12]^

Near visual tasks affect various eye parameters such as anterior chamber depth,^[2,13,14,15]^ vitreous chamber depth,^[13]^ intraocular pressure,^[16,17,18]^ lens thickness,^[13]^ axial length of the eye,^[2,13]^ retina,^[19]^ choroid,^[15]^ sclera,^[20,21]^ as well as eyelid pressure.^[22,23,24]^ There are mixed results on the effects of near visual tasks on corneal parameters.^[23,24,25,26,27]^ Despite the lack of changes in corneal topography after the act of reading in some studies,^[25]^ there are reports suggesting monocular diplopia due to cornel distortion induced by eyelid pressure in downward gaze after this activity.^[28,29]^ Changes in eye aberrations have been reported during reading in people with a smaller palpebral fissure.^[13]^ Change of central corneal curvature was also found 40 minutes after instillation of topical pilocarpine 4%, which simulates accommodation irrespective of the reading position in near visual tasks.^[30]^ Because near visual tasks are usually performed in the downward gaze, the effect on corneal refractive parameters may be related to upper eyelid pressure in downward gaze during near visual tasks.^[2,3,26,27]^ The recovery time of corneal changes following the reading activity has been found to be significantly related to the duration of reading, and a higher recovery time has been seen after long study periods.^[27]^

Today, near visual work with digital devices has increased. Many cases of prolonged near visual work are related to, first, office staff who use computers in front of them without downward gaze and, second, young people who spend a lot of time watching videos. Therefore, this study aimed to evaluate the effects of near visual work in the form of watching a movie on a computer screen, which is one of the most common near visual tasks, in straight gaze position while avoiding the downward gaze (to eliminate the role of eyelid force caused by downward gaze during near work) on corneal topographic parameters among young adults.

**Figure 1:**
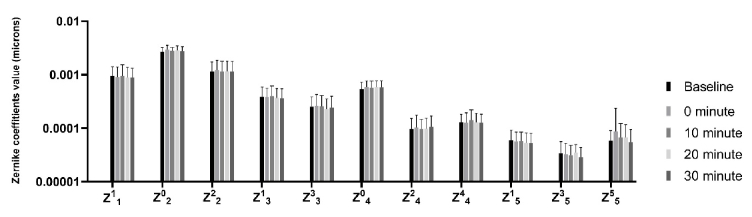
Mean and standard deviation of Zernike coefficients before and after watching the movie.

**Table 1:** Demographic and baseline measurement variables


**Variable**	**Values (mean ± SD, 95% CI)**
Age (yr)	24.10 ± 4.13 (22.70 to 25.53)
Gender (no)	28 female/2 male
Laterality of the dominant eye	23 right eyes/7 left eyes
Spherical refractive error (diopter)	–2.10 ± 1.06 (–2.49 to –1.75)
Astigmatism (diopter)	–0.31 ± 0.24 (–0.39 to –0.23)
Intraocular pressure (mmHg)	18.11 ± 0.89 (17.1 to 19.19)
Pupil size (mm)	3.49 ± 0.69 (3.2 to 3.76)
	
	
white<bcol>2</ecol>CI, confidence interval; mm, millimeter; SD, standard deviation; yr, years

**Table 2:** Mean and standard deviation of refractive parameters and eccentricity before and after watching the movie


**Variable**	**Before watching the movie**	**After watching the movie**				***P*** -value ${\;}^{*}$ * *
		**Immediate**	**10 minutes**	**20 minutes**	**30 minutes**	* *
Corneal apex power (diopter)	44.32 ± 2.04	44.41 ± 2.04	44.34 ± 2.04	44.40 ± 2.03	44.37 ± 2.03	0.477
Steepest corneal curvature (diopter)	44.43 ± 2.03	44.46 ± 2.03	44.44 ± 2.03	44.44 ± 2.06	44.47 ± 2.04	0.717
Steepest corneal axis (degree)	91.04 ± 19.25	89.26 ± 16.44	85.32 ± 20.41	88.35 ± 18.55	90.49 ± 13.55	0.439
J0	–0.003 ± 0.057	0.002 ± 0.062	0.002 ± 0.058	0.005 ± 0.065	0.012 ± 0.059	0.834
J45	–0.008 ± 0.056	–0.018 ± 0.048	–0.013 ± 0.052	0.003 ± 0.047	–0.015 ± 0.047	0.534
Peripheral astigmatism (diopter)	0.21 ± 0.14	0.25 ± 0.17	0.23 ± 0.15	0.21 ± 0.16	0.25 ± 0.16	0.179
Peripheral astigmatism axis (degree)	71.41 ± 63.29	85.90 ± 65.53	96.21 ± 66.98	74.07 ± 65.40	108.31 ± 60.91	0.082
Mean corneal eccentricity	0.51 ± 0.12	0.52 ± 0.08	0.52 ± 0.08	0.52 ± 0.09	0.52 ± 0.09	0.702
Spherical eccentricity	0.53 ± 0.08	0.52 ± 0.8	0.51 ± 0.9	0.52 ± 0.9	0.51 ± 0.9	0.270
Maximum decentration (mm)	0.37 ± 0.26	0.35 ± 0.19	0.33 ± 0.18	0.33 ± 0.18	0.33 ± 0.23	0.553
Axis of maximum decentration (degree)	205.17 ± 89.27	179.28 ± 93.45	203.93 ± 87.09	213.10 ± 82.86	189.90 ± 95.98	0.186
Irregularities	0.045 ± 0.013	0.044 ± 0.017	0.042 ± 0.010	0.040 ± 0.012	0.042 ± 0.013	0.208
	
	
${\;}^{*}$ *P*-value for repeated measures ANOVA before, immediately, at 10, 20, and 30 minutes intervals after watching the movie						

**Table 3:** Mean and standard deviation of asphericity of the quadrants at angles 10,º 15º, 20º, 25º, and 30º before and after watching the movie


**Variable**	**Before watching the movie**	orange**After watching the movie**				P-value ${\;}^{*}$
		**Immediate**	**10 minutes**	**20 minutes**	**30 minutes**	
Asphericity of the quadrants at 10 ${\;}^{\circ }$	–0.191 ± 0.116	–0.165 ± 0.139	–0.198 ± 0.114	–0.195 ± 0.092	–0.188 ± 0.113	0.296
Asphericity of the quadrants at 15 ${\;}^{\circ }$	–0.215 ± 0.10	–0.189 ± 0.125	–0.210 ± 0.096	–0.215 ± 0.097	–0.206 ± 0.099	0.163
Asphericity of the quadrants at 20 ${\;}^{\circ }$	–0.230 ± 0.102	–0.207 ± 0.125	–0.218 ± 0.092	–0.230 ± 0.110	–0.217 ± 0.101	0.291
Asphericity of the quadrants at 25 ${\;}^{\circ }$	–0.270 ± 0.098	–0.240 ± 0.136	–0.259 ± 0.093	–0.263 ± 0.104	–0.262 ± 0.101	0.255
Asphericity of the quadrants at 30 ${\;}^{\circ }$	–0.300 ± 0.095	–0.273 ± 0.164	–0.292 ± 0.097	–0.293 ± 0.102	–0.296 ± 0.103	0.417
	
	
${\;}^{*}$ *P*-value for repeated measures ANOVA before, immediately, at 10, 20, and 30 minutes intervals after watching the movie						

**Table 4:** Mean and standard deviation of keratoconus diagnostic indices before and after watching the movie


**Variable**	**Before watching the movie**	orange**After watching the movie**				P-value ${\;}^{*}$
		**Immediate**	**10 minutes**	**20 minutes**	**30 minutes**	
ISV	18.50 ± 5.18	18.66 ± 4.63	18.28 ± 5.53	18.03 ± 5.11	17.93 ± 5.23	0.408
IVA	0.111 ± 0.05	0.107 ± 0.04	0.111 ± 0.05	0.105 ± 0.04	0.106 ± 0.04	0.559
KI	0.014 ± 0.014	0.014 ± 0.015	0.015 ± 0.017	0.015 ± 0.016	0.015 ± 0.014	0.684
CKI	1.007 ± 0.004	1.007 ± 0.005	1.006 ± 0.005	1.006 ± 0.004	1.006 ± 0.005	0.097
Rmin	7.493 ± 0.340	7.480 ± 0.345	7.496 ± 0.348	7.484 ± 0.356	7.496 ± 0.347	0.589
IHA	6.12 ± 4.63	5.61 ± 3.60	6.13 ± 4.64	5.66 ± 3.91	5.67 ± 3.34	0.564
IHD	0.0046 ± 0.0023	0.0043 ± 0.0025	0.0046 ± 0.0027	0.0042 ± 0.0025	0.0044 ± 0.0021	0.654
	
	
ISV**, **index of surface variance; IVA, index of vertical asymmetry; KI, keratoconus index; CKI, central keratoconus index; Rmin,minimum sagittal curvature; IHA,index of height asymmetry; IHD,index of height decentration ${\;}^{*}$ *P*-value for repeated measures ANOVA before, immediately, at 10, 20, and 30 minutes intervals after watching the movie						

##  METHODS

This study was conducted on optometry students of Mashhad University of Medical Sciences. The study protocol was reviewed and approved by the Ethics Committee of Mashhad University of Medical Sciences (IR.MUMS.FHMPM.REC.1400.021). All participants signed the informed consent form.

The recruited subjects were comprised of myopic adults with the refractive error of –0.5 to –5 D, astigmatism ≤ 1 D, tear break-up time (TBUT) ≥ 10 s, visual acuity (VA) ≥ 20/20, and normal binocular vision. The exclusion criteria were the presence of any systemic diseases, history of using hard contact lenses, wearing soft contact lenses in the past two weeks before the study,^[31]^ history of eye surgeries, presence of eyelid abnormalities, presence of any irregularity in the cornea (e.g., pinguecula, pterygium, corneal ectasias, and keratoconus), and the use of drugs that affect dry eyes.

First, a questionnaire about the subjects' history of systemic and eye diseases, contact lens use, medications, and eye surgeries was filled out. Then, complete eye examinations including slit lamp and ophthalmoscopy, non-contact tonometry (Computerized Tonometer CT-20), and the non-invasive tear break-up time (NIBUT) test were performed. The refractive errors of all subjects were identified with an auto-refractometer (AR-610, Nidek Co, Ltd., Tokyo, Japan), and their subjective refraction and best corrected visual acuity for distance were determined.

The subjects were asked not to perform any lengthy reading, computer work, or other sustained visual tasks, starting from the night before the study.

The dominant eye of the subjects was examined by Oculus Keratograph 4 (OCULUS, Wetzlar, Germany), which provides repeatable measurements of corneal topography.^[32]^ Then, the subjects with the best distance correction watched a single movie for 30 minutes from a laptop (LENOVO YOGA 300, 11.6 inches, resolution of 1366 × 768), which was placed at a distance of 40 cm from their eyes. To prevent the subjects' downward gaze while watching the movie, we adjusted the height of the monitor according to the lateral canthus of the subjects so that the eyes were aligned with the center of the monitor. Participants were also asked to blink normally while watching the film. Corneal topography of the dominant eye was performed immediately (0 min) after watching the movie, as well as at 10, 20, and 30 minutes intervals after the end of the movie. During this period, the subjects did not do any near visual work.

All topography measurements were done in a dark room after the participant's a few blinks to make the tear layer even and increase the accuracy of the test. This was accomplished by adjusting the position of the target in the center of the participant's pupil while he/she was looking at the yellow target of the device. Mire uniformity reflected from the cornea was examined to evaluate the image quality; the measurement was repeated if the quality was not appropriate.

The temperature, humidity, and illumination of the room at the time of watching the movie and at the recovery time were stable and identical for all participants (27ºC, 0 gm ${\;}^{-3}$ and 260 lux). All measurements were performed at 8 to 11 AM for all participants in accordance with the daily changes of the corneal topography and aberrations.^[33]^

The recorded indices and parameters included:

Zernike coefficients, derived from a 5-mm pupil diameter using Zernike polynomials up to the fifth radial order.

Asphericity, refractive parameters, and Fourier indices: spherical eccentricity, mean corneal eccentricity (Ecc), maximum decentration and its axis, irregularities, corneal apex power (Apex), steepest corneal curvature (Ks), steepest corneal axis (Ks axis), peripheral astigmatism and its axis, J0 and J45 vector components (central astigmatism values were converted to vector components using vector analysis), and asphericity of quadrants at angles 10º, 15º, 20º, 25º, and 30º.

Diagnostic indices of keratoconus: index of vertical asymmetry (IVA), index of surface variance (ISV), keratoconus index (KI), central keratoconus index (CKI), index of height asymmetry (IHA), index of height decentration (IHD), and minimum sagittal curvature (Rmin).

Statistical analysis was performed in SPSS (version 15; Chicago, SPSS Inc) and the plotted figure was drawn with Graph Pad Prism (version 6.07 for Windows; Graph Pad software. Inc). Repeated-measures of the one way analysis of variance (ANOVA) test were used to probe any potential effect of the near visual task on corneal parameters in the subjects by comparing results before watching the movie immediately and at 10, 20, and 30 minutes intervals after watching the movie. *P* < 0.05 was considered statistically significant.

##  RESULTS

Overall, 30 people (28 women and 2 men) aged 20–32 years (25.10 ± 4.13 years) participated in this study. Table 1 shows the mean and standard deviation of refractive errors, intraocular pressure, and pupil size in the studied subjects.

Figure 1 shows the mean and standard deviation of Zernike coefficients before and after the near visual task. Repeated-measures of ANOVA revealed that watching the movie had no effect on Zernike coefficients (*P* > 0.130). Likewise, the paired *t*-test with Bonferroni correction showed that the Zernike coefficients before watching the movie were not significantly different from the values obtained immediately (*P* > 0.061), at 10 minutes (*P* > 0.238), 20 minutes (*P* > 0.204), and 30 minutes after watching the movie (*P* > 0.111).

Table 2 presents the mean and standard deviation of eccentricity, asphericity, and corneal refractive parameters before and after watching the movie. It appears that watching the movie had no effect on irregularities (*P *= 0.208), spherical eccentricity (*P *= 0.270), maximum decentration (*P* = 0.553), axis of maximum decentration (*P *= 0.186), Ecc (*P *= 0.702), apex (*P *= 0.552), Ks (*P *= 0.717), Ks axis (*P *= 0.439), J0 (*P *= 0.834), J45 (*P *= 0.534), peripheral astigmatism (*P *= 0.179), and peripheral astigmatism axis (*P *= 0.082)

Table 3 shows the mean and standard deviation of asphericity of quadrants at angles 10º, 15º, 20º, 25º, and 30º before and after watching the movie. Accordingly, watching the movie had no effect on the average asphericity of the quadrants at 10º (*P *= 0.296), 15º (*P *= 0.163), 20º (*P *= 0.291), 25º (*P *= 0.255), and 30º (*P *= 0.417).

Table 4 shows the mean and standard deviation of keratoconus diagnostic indices before and after watching the movie. It can be seen that watching the movie had no effect on ISV (*P *= 0.408), IVA (*P* = 0.559), KI (*P *= 0.684), CKI (*P *= 0.097), Rmin (*P *= 0.589), IHA (*P *= 0.564), and IHD (*P *= 0.654).

##  DISCUSSION

This study investigated the effect of near visual task (watching a movie for 30 minutes) in the straight gaze position on corneal topographic parameters. Our results did not show a significant difference in the measured parameters of the cornea before, immediately, and 10, 20, and 30 minutes after watching the movie.

The results indicated that the implemented near visual task had no effect on Zernike coefficients. Previous studies have pointed to different results concerning the effect of near visual work on aberrations.^[34,35,36]^ Some studies have reported an increase in aberrations after near visual work.^[26,34,35,37]^ Meanwhile, it has been found that near visual tasks such as reading and microscopy generally induce greater changes in corneal wave-front compared with computer work.^[36]^ The reason for the difference in the results of these studies can be attributed to the difference in the type and duration of near visual tasks applied in each research. In two studies by Buehren^[26]^ and Vasudevan,^[35]^ who observed an increase in aberrations in emmetropic and myopic subjects after reading, the duration of the reading task was 2 hr and 1 hr, respectively, which are more than the duration of near visual work in our study. On the other hand, these two studies were carried out under downward gaze conditions (viewing angle of 60º and 30º in Buehren^[26]^ and Vasudevan^[35]^ studies, respectively), while in our study watching the movie occurred in the straight gaze position. It is worth noting that downward gaze causes greater changes due to the greater pressure the eyelids exert on the cornea,^[22,23,24][36]^ because eye aberrations have been shown to increase from primary gaze to downward gaze.^[3]^ It was also found that long-term near visual tasks such as reading or computer use can reduce the quantity and quality of the tear film,^[38,39]^ and changes in tear film thickness and its irregularity can cause additional aberrations.^[40]^ In this study, although the stability of the tear film during near work was not investigated, changes in tear film stability might not have occurred due to the shorter duration of near visual work and reminding participants to blink frequently while watching the film.

The results of this study showed no change on corneal refractive parameters and corneal astigmatism before and after the near visual task, which is consistent with some of the previous studies.^[2,25]^ Ghosh reported no change in corneal refractive power in primary gaze when compared to downward gaze (25º) in emmetropic and myopic subjects.^[2]^ Also, Chen et al concluded that near visual work does not cause specific topographic changes in emmetropic or myopia groups.^[25]^ On the other hand, some studies have suggested significant changes in corneal power after near visual work.^[14,26,27,41]^ Collins reported changes in corneal power in emmetropic and myopic subjects after even 10 minutes of reading; the author attributed these changes to eyelid pressure caused by downward gaze during reading.^[27]^ Eyelid pressure during near visual work seems to have been proposed in regard to the effect of this activity on astigmatism.^[42]^ In our study, this factor was diminished because the near visual task was performed in the straight gaze position. Comparing the results of the present study with previous studies implies that eyelid pressure probably plays a more important role in causing corneal refractive changes than does the duration of visual task. Indeed, in some previous studies,^[2,22,24,27]^ more changes were observed despite the shorter duration of near visual work when compared to our research, which could be due to the difference in gaze direction. Another possible reason for the incompatibility between our results and the results of other studies is the type of defined task: the activity of reading requires more pursuit and saccadic eye movements than that required when watching movies. In fact, there is evidence to support that eye movements cause greater changes in cornea parameters than fixation.^[22]^

Our results showed that watching movies has no effect on keratoconus indices and the mean asphericity of the quadrants in different angles. Asphericity refers to the rate of curvature change along the corneal surface from the apex to the peripheral regions.^[43]^ Considering that in the present study there were no significant changes in the corneal refractive and asphericity parameters after watching the movie, it seems logical to keep the keratoconus indices constant.

This study had some limitations. One of the limitations was the lack of a control group to control the confounding factors. Another was the higher number of women than men, so it was not possible to compare the changes in terms of gender. It is recommended to conduct further studies with a larger sample size in the presence of a control group, and to measure corneal biomechanical factors along with topographical parameters.

The results of the present study showed that watching movies has no effect on corneal topographic parameters and Zernike coefficients. It should be stressed, however, that this is the case only when the near visual work takes place in the straight gaze and no other gaze directions.

##  Financial Support and Sponsorship

None.

##  Conflicts of Interest

None.
